# Identification of Osteosarcoma-Related Specific Proteins in Serum Samples Using Surface-Enhanced Laser Desorption/Ionization-Time-of-Flight Mass Spectrometry

**DOI:** 10.1155/2014/649075

**Published:** 2014-04-24

**Authors:** Jianli Gu, Jitian Li, Manyu Huang, Zhiyong Zhang, Dongsheng Li, Guoying Song, Xingpo Ding, Wuyin Li

**Affiliations:** ^1^Luoyang Orthopedic-Traumatological Hospital, Luoyang Institute of Orthopedic and Traumatology, Henan Province, Luoyang 471002, China; ^2^Department of Biological Sciences, The University of Texas at El Paso, El Paso, TX 79968, USA

## Abstract

Osteosarcoma (OS) is the most common malignant bone tumor. To identify OS-related specific proteins for early diagnosis of OS, a novel approach, surface-enhanced laser desorption/ionization-time-of-flight mass spectrometry (SELDI-TOF-MS) to serum samples from 25 OS patients, 16 osteochondroma, and 26 age-matched normal human volunteers as controls, was performed. Two proteins showed a significantly different expression in OS serum samples from control groups. Proteomic profiles and external leave-one-out cross-validation analysis showed that the correct rate of allocation, the sensitivity, and the specificity of diagnosis were 100%. These two proteins were further identified by searching the EPO-KB database, and one of the proteins identified as Serine rich region profile is involved in various cellular signaling cascades and tumor genesis. The presence of these two proteins in OS patients but absence from premalignant and normal human controls implied that they can be potential biomarkers for early diagnosis of OS.

## 1. Introduction 

Osteosarcoma (OS), characterized by the production of osteoid material by malignant osteoblastic cells, is a primary malignant bone tumor deriving from primitive bone-forming mesenchymal cells [1]. Overall, the treatment strategies are preoperative chemotherapy and surgical resection followed by postoperative chemotherapy and adjuvant therapy for several years [2]. The 5-year survival rates for patients with localized osteosarcoma have dramatically improved from less than 15% in the 1950s to greater than 60% since the 1980s [3]. Despite advances in diagnostic and treatment regiments, progress since the 1980s has been minimal with 5-year survival rates still in the 60–70% range [4]. One of the reasons could be the presence of mutated oncogenes, which confer drug resistance of the cancer cell.

Currently, the diagnosis of OS is generally dependent on a comprehensive examination including clinical symptoms, imaging, laboratory examinations, biopsy, and immunohistochemistry. Clinically, OS was always diagnosed at the middle or even at a late stage. Therefore, the long-term survival rate of OS has not been improved in the past 20 years [2]. The histological examination of the biopsy specimens is still preferred by many orthopedic oncologists for the diagnosis of OS. Despite its invasive procedure, the accuracy of diagnosis may vary among different sample collection and different observers, making the clinical prediction questionable. Thus, we have focused our attention on the development of a noninvasive method for the early diagnosis of osteosarcoma.

Many studies have identified that antigenic changes in cells can be recognized by the immune system of patients. Some early studies demonstrated the presence of serum autoantibodies to a panel of known tumor associated antigen (TAA) in various human cancers [5]. The study indicated that autoantibody reactivity to individual TAA rarely exceeded 20% in the cancer patient populations compared with normal human sera (NHS) which are usually less than 5%. It is conceivable that specific autoantibody profiles can be identified with help for discriminating autoantibody reactivity between cancer patients and control individuals and distinguishing between some types of cancer. So far, only a few studies have been performed in the field of using autoantibodies as diagnostic markers in osteosarcoma.

Several different approaches based on mass spectrometry (MS) have been applied in the search for cancer biomarkers [6]. Recently, technological improvements in MS have greatly increased their exploits in biomarker discovery. Direct analysis of serum samples using MS is becoming more popular due to its high-throughput nature and increased sensitivity. One of these proteomic approaches, surface-enhanced laser desorption/ionization-time-of-flight mass spectrometry (SELDI-TOF-MS), is a rapid and sensitive proteomic technique to identify biomarkers in various forms of cancers [7, 8]. This technology has been effectively used in the validation of serum antigens for early-stage detection of various cancers, such as prostate [9], ovarian [10], and breast cancers [11]. Therefore, exploitation of SELDI-TOF-MS for screening biomarkers in OS is applicable. The searching database tool, empirical proteomic ontology knowledge base (EPO-KB), including tens of thousands of mass to charge ratio (*M*/*Z*) protein function, was used to identify proteins characterized from SELDI-TOF-MS. Using SELDI-TOF-MS combined with EPO-KB makes it possible to directly separate and identify the proteins from human osteosarcoma sera [12]. The major aim of this study using SELDI-TOF-MS combined with EPO-KB is to identify serum markers from human osteosarcoma sera as potential OS-associated markers for early diagnosis of OS.

## 2. Materials and Methods

### 2.1. Serum Samples

Sera from 25 Chinese patients, Han ethnic (fourteen males and eleven females, mean age 19.3 years, and range 2–50), with high-grade OS were collected at the time of diagnosis before biopsy and chemotherapy were analyzed. All samples were provided by Luoyang Orthopedic-Traumatological Hospital and Luoyang Institute of Orthopedic and Traumatology between Jan. 2008 and Dec. 2010. We randomly collected 26 human serum samples from normal age-matched donors in the same hospital as a normal control and 16 samples from osteochondroma (OC) (nine males and six females, mean age 16.8 years, and range 4–48 years, Chinese patients, Han ethnic) as benign tumor control. All blood samples (5 mL from each patient or normal person) were collected in EDTA-containing tubes at room temperature and immediately centrifuged at 1000 rpm for 10 min. The serum supernatant was collected and divided into aliquots and stored at −80°C. The study was approved by the ethics committee of Luoyang Orthopedic-Traumatological Hospital and Luoyang Institute of Orthopedic and Traumatology. The written informed consent for participation was obtained from all subjects.

### 2.2. Sample Preparation

Serum samples were diluted to a certain concentration and immediately centrifuged (20,000 rpm) for 10 min at 4°C. Twenty microliters of each serum was denatured by adding 40 mL of U9 buffer containing 9 mol/L uric acid, 2% CHAPS (pH 9.0), and 50 mmol/L Tris-HCl (pH 9.0) and shaken for 20 min at 4°C. Twenty microliters of the denatured samples were fractionated by 240 *μ*L of U1 buffer and shaken for 30 min. All these samples were used in proteomic profiling.

### 2.3. SELDI-TOF-MS and Database Search

SELDI-TOF-MS was performed on NP20 chips. Each chip was added with bluestone (50 *μ*L, 100 mmol/L) and shaken (200 rpm) for 5 min at room temperature, and the bluestone solution was immediately poured out. After being washed using ionized water for 5 times, the chip was added with 50 *μ*L of CM Low Stringency Buffer (0.1 M sodium acetate, pH 4.0), shaken (200 rpm) for 5 min. Then, each chip was added with 150 *μ*L of standard buffer containing 100 mol/L sodium phosphate and 500 mmol/L sodium chloride (pH 7.0) and incubated on the oscillator for 5 min at room temperature, and the buffer solution was removed. This process was repeated one more time. Each serum sample (50 *μ*L) was randomly assigned to a spot on each protein-chip array and incubated on the oscillator for 60 min at 4°C. The samples were denatured by 150 *μ*L of standard buffer, washed and oscillated for 3 times each for 5 min, and quickly washed with 1 mmol/L HEPES (pH 7.0). Chip EAM (sinapinic acid (SPA)) was added with 100 mmol/L acetonitrile (75 *μ*L) and trifluoroacetic acid (TFA) (75 *μ*L). The dissolved SPA was centrifuged for 1 min. 2 *μ*L of SPA was spotted twice, 1 *μ*L each time. The chip was air-dried before spotting. The reagents such as uric acid, acetonitrile, TFA, and SPA were purchased from Sigma. The PBSIIc and IMAC3-Cu were calibrated externally by using the all-in-one peptide and protein II molecular mass standards (Ciphergen Biosystems). Data were collected with Ciphergen protein-chip software. Proteins were randomly spotted on the chip. Each sample was analyzed in duplicate to minimize the effects of intra-assay variation. Proteomic profiles on the protein-chip arrays were detected by a Protein Biology System (Model PBSIIc).

### 2.4. Statistical Analysis

Data analysis was performed initially with Ciphergen Express Software 3.0 (Ciphergen Biosystems). Statistical signification peaks were recorded by $\overset{-}{x}\pm s$ and identified by using parametric *t*-test with *P* value of 0.001 with Biomarker Wizard and Biomarker Pattern System software. The diagnostic accuracy was measured by external leave-one-out cross-validation, which is one of the feature selection steps. Other statistical analyses were performed using SPSS 11.5.

## 3. Results

### 3.1. Identification of Two Statistically Significant Proteins of OS

**Figure 1:**
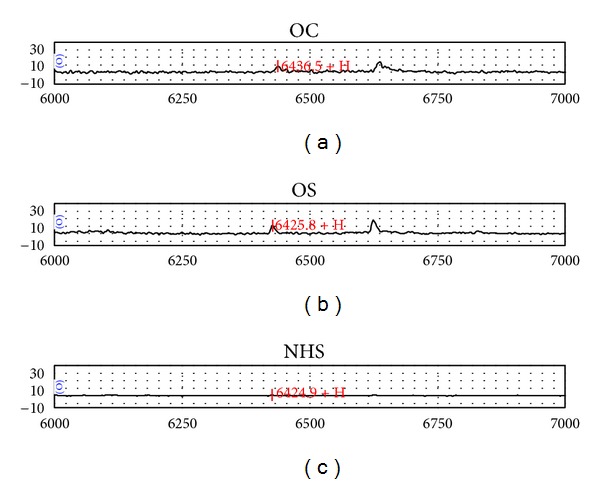
The SELDI-TOF-MS profiles of osteochondroma (OC), osteosarcoma (OS), and normal human serum (NHS) at the 6438 Da. The SELDI-TOF-MS profiles of the serum samples from OC (a), OS (b), and NHS (c).

**Figure 2:**
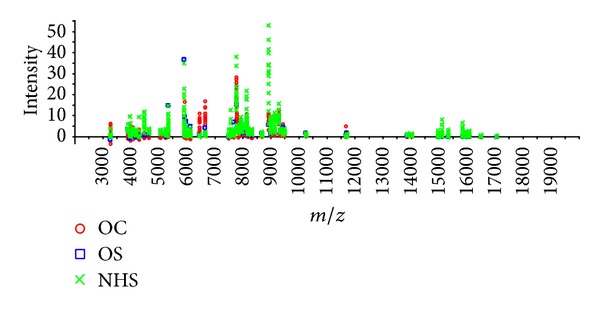
The protein intensity of osteochondroma (OC), osteosarcoma (OS), and normal human serum (NHS). The protein intensity of the serum samples from NHS (green panel), OS (blue panel), and OC (red panel).

Sixty-seven serum samples were assayed by SELDI-TOF-MS. From a total of 67 serum samples, 25 serum samples are from OS patients compared with the control group of 42 serum samples (16 from osteochondroma and 26 from normal human serum). Interestingly, two protein peaks differed significantly in the OS patient group. One protein peak at 3954 Da was overexpressed and another one at 6438 Da (Table 1). The mass spectrum shows the comparison of three serum samples from patients with OS and OC as well as a serum from a normal individual (Figures 1 and 2).

### 3.2. Classification Tree Topology for GROUP and Analysis of Specificity and Sensitivity

**Figure 3:**
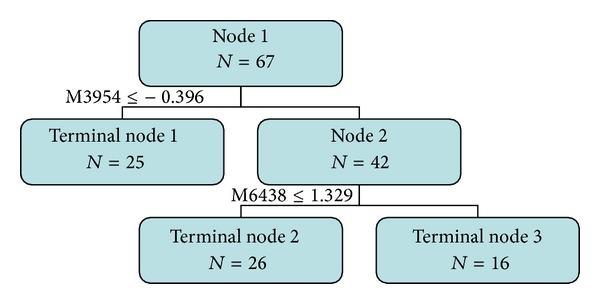
Classification tree topology for GROUP. Node 1 total sample (25 OS, 26 normal, and 16 OC), M3954 ≤ −0.396 (including 25 OS) divided to terminal node 1; M3954 > −0.396 (including 26 normal and 16 OC) divided to node 2; M6438 ≤ 1.329 (including 26 normal) divided to terminal node 2; M6438 > 1.329 (including 16 OC) divided to terminal node 3.

In order to take a marked contrast, we apply the Biomarker Pattern software to analyze protein differences in the template group to *m*/*z*, respectively, 3954 Da and 6438 Da proteins composed of two different diagnostic classification tree models (Figure 3). The 67 samples were repeated sampling in the learning mode, and the diagnostic sensitivity was 98.51% and specificity was 98.51%, while, in test mode, the diagnostic sensitivity was 98.51%, and specificity was 100.00%. It was verified by leave-one-out and 3-fold cross-validation that correct grouping rate was 100% (67/67), good response rate was 100% (25/25), and specificity was 100% (42/42).

### 3.3. Database Search Results

We searched the *M*/*Z* of 3954 Da and 6438 Da in the first classification tree models and got related results in the EPO-KB database. The relevant information of 6438 Da protein was retrieved; however, the 3954 Da protein needs further identification (http://www.expasy.org/cgi-bin/prosite-search-ac?PDOC50099 and http://us.expasy.org/prosite/PS50324). It is serine rich protein sequences involved in cell signal transduction and carcinogenesis. In some microtubule plus end tracking proteins, there are some basic amino acid serine rich sequences. These basic amino acids serine rich sequences are often mediated by the microtubule plus end tracking proteins with microtubules and EB protein and the role of other proteins. Protein peak of *M*/*Z* at 6438 Da in the B-cell lymphoma is highly expressed and, in the low expression of T cell lymphoma, it is closely related to the human immune system regulation.

## 4. Discussion 

SELDI-TOF-MS is a relatively new proteomic technology that has impacted many areas of biological research [13, 14]. The high-throughput nature of this technology allows the processing of many samples simultaneously in a relatively short period of time. It requires only small amounts of samples for profiling, which makes it particularly suitable for clinical or translational studies. SELDI-TOF-MS has been successfully applied in identifying early detection biomarkers in multiple cancers, including ovarian, prostate, and breast cancers. However, SELDI-TOF-MS technology is not widely used in bone oncology to identify biomarkers and perform molecular classification, partly due to the limited sample size when compared to other cancers. The current study shows that patients with OS and OC and normal individuals can be distinguished from each other by the special proteins with different *M*/*Z* characters screened by SELDI-TOF-MS. The results showed three samples determined by the difference of the following standards (M3954 ≤ −0.396 (normal), M6438 ≤ 1.329 (OS), and M6438 > 1.329 (OC)). It was verified by leave-one-out and 3-fold cross-validation that correct grouping rate was 100% (67/67), which proved that the results is reliable.

The diagnosis of OS generally depended on a comprehensive way including clinical symptoms, signs, image, laboratory examinations, biopsy, immunohistochemistry, and other methods. The surgical biopsy is also the terminal way for final diagnosis, which is an invasive procedure and has risks of enhancing tumor dissemination and metastases. If we can perform specific markers with high sensitivity and specificity using blood specimens from the patients, it will be more convenient and noninvasive to the patients at the initial diagnosis. These specific markers combined with X-ray exam could be used to monitor OS development in coordination with chemotherapy or radiography. And this combination would greatly improve the early-stage detection and treatment of OS. The present study has found the *M*/*Z* of 3954 Da and 6438 Da proteins in the classification tree models and gotten 6438 Da in the EPO-KB database, which is a serine rich protein sequences involved in cell signal transduction and carcinogenesis. These basic amino acids serine rich sequences are often mediated by the microtubule plus end tracking proteins with microtubules and EB protein and the role of other proteins. Since protein peak of M/Z at 6438 Da showed high expression in the B-cell lymphoma and low expression in T-cell lymphoma, it is closely related to the human immune system regulation. We thought that the 6438 Da protein identified by SELDI-TOF-MS may potentially be used for early detection, diagnosis, therapeutically monitoring, and prognosis of OS. In addition, randomized clinical trials are required to investigate its effect (or lack thereof).

SELDI-TOF-MS technology is still more constraining in clinical research, because the only result we can get is *m*/*z*, rather than the molecular weight. After finding the different proteins by SELDI-TOF-MS, further studies with chromatography, two-dimensional electrophoresis, spectroscopy, and other techniques need to be performed to identify these proteins immensely. However, these laboratory approaches could not be used for large-scale screening of biomarkers in clinical application. Additionally, its wide acceptance is still in question due to some concerns regarding reproducibility and reduced sensitivity at high molecular weight range [15].

These findings suggest that the *M*/*Z* of 3954 Da and 6438 Da proteins could potentially be biomarkers for early diagnosis of osteosarcoma. It would be interesting and important to conduct other independent studies in large populations for comparison. Future functional studies are warranted to verify these findings and to improve our understanding of the underling molecular mechanism of genetic contribution to OS carcinogenesis.

## Figures and Tables

**Table 1:** The *M*/*Z*s of 2 special serum proteins in three groups.

*M*/*Z*	NHS (mean ± SD)	OS (mean ± SD)	OC (mean ± SD)	*P* value	VI (score)
3954.00 Da	1.75 ± 0.35	−0.78 ± 0.08	1.47 ± 0.35	0.001	100
6438.00 Da	0.45 ± 0.21	0.88 ± 0.13	5.78 ± 1.12	0.001	100

*M*/*Z*: mass to charge ratio; NHS: normal human serum; OS: osteosarcoma; OC: osteochondroma; VI: variable importance.
